# Supplementary figures and images for: The Genomic Landscape of Urothelial Carcinoma with High and Low ERBB2 Expression
Source: Cancers (Basel). 2023 Dec 6;15(24):5721. doi: 10.3390/cancers15245721 (PMC10742086; doi:10.3390/cancers15245721)

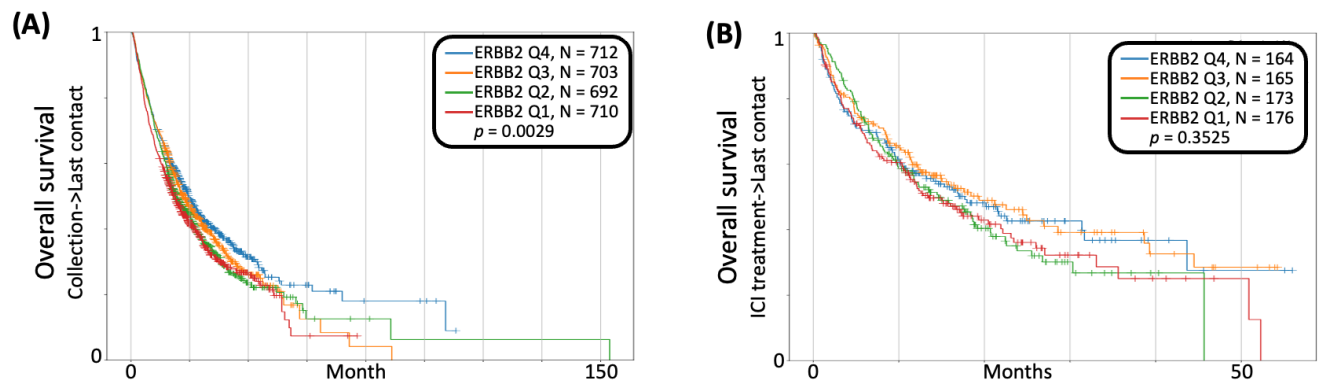

**Figure S1. (A)** OS for *ERBB2*-high vs *ERBB2*-low tumors. **(B)** Survival since treatment with ICI.

Supplement: Supplementary file 1 [file cancers-15-05721-s001.zip › cancers-2721473-supplementary.pdf]
